# P388: Evaluation of the outsourcing of public hospital cleaning / case of university hospital Yalgado Ouedraogo

**DOI:** 10.1186/2047-2994-2-S1-P388

**Published:** 2013-06-20

**Authors:** J Zoungrana, A Traoré

**Affiliations:** 1 RIPAQS, Burkinabe Association for Improving Patient Safety, Burkina Faso; 2 Hospital Universitary Centre Yalgado Ouedraogo, Ministry of Health, Ouagadougou, Burkina Faso

## Introduction

Lack of tangible evidence proving the effectiveness and efficiency of the outsourcing of cleaning compared to self cleaning. Need to evaluate the outsourcing of cleaning.

## Objectives

Evaluate cleaning subcontracting of CHUYO. The specific objectives were: 1. To assess the cleaning process at CHUYO; 2. To assess the effectiveness of cleaning in comparison to standards; 3. To discuss the factors explaining any gaps identified.

## Methods

Descriptive cross-sectional survey conducted from 04 to 10 July 2011. Collection of information from patients and healthcare personnel. We used a questionnaire survey addressing the following items: work organization, human resources, cleaning procedures, material resources. Surface sampling 15 minutes after cleaning operations were conducted.

## Results

- 95% of patients feel that they were satisfied with the cleaning done by a private company;

- 75% of patients feel that they are not disturbed by the passage cleaning crews;

- 89% of patients report that their products used do not cause any discomfort;

- 77% of professionals are satisfied with the quality of cleaning, - There were dissatisfaction in 3 points: transit schedules not suitable for some patients, lack of staff, staff discourteous.

- 93% of study participants prefer subcontracted cleaning rather than self cleaning.

## Conclusion

Strengthening the achievements of subcontracting (apparent cleanliness and satisfaction of users and health professionals). Ensure training of cleaning staff, establish mechanisms for monitoring bacteriological quality surfaces and disinfectants.

## Disclosure of interest

None declared

